# Validation of a novel magnetic resonance imaging classification and recommended treatment for lateral elbow tendinopathy

**DOI:** 10.1186/s12891-022-05758-z

**Published:** 2022-08-22

**Authors:** Panithan Tuntiyatorn, Rachaporn Taweesakulvashra, Thepparat Kanchanathepsak, Chanakarn Rojpitipongsakorn, Tulyapruek Tawonsawatruk

**Affiliations:** 1 Faculty of Medicine, Chakri Naruebodindra Medical Institute, Ramathibodi Hospital, Mahidol University, Bangkok, Thailand; 2 Department of Orthopaedics, Faculty of Medicine, Ramathibodi Hospital, Mahidol University, Bangkok, Thailand

**Keywords:** Lateral epicondylitis, Magnetic resonance imaging, Elbow tendinopathy

## Abstract

**Background:**

Lateral epicondylitis is one of the most common upper extremity problems presented to orthopedic surgeons. Despite a rapid and accurate arrival at a diagnosis by clinical examination, there exists no consensus classification for this condition, which hampers clinical approaches for treatment of the disease based on its severity. Thus, the aim of this study was to propose and valiadate a new magnetic resonance imaging (MRI) classification of lateral epicondylitis, staging by tendinosis, the degree of thickness tears of the common extensor tendon (CET) and bone bruise lesion.

**Method:**

MRI assessment of the elbow of 75 patients (57 women and 18 men; mean age:51.4 years (range,34–73) from Jan 2014 to Jan 2021 who were diagnosed with lateral epicondylitis were included in the study. MR images were reviewed retrospectively by two independent upper extremities orthopedists and one musculoskeletal radiologist. Inter- and intra-observer reliabilities for the classification were calculated using kappa statistics for the analysis of interrater agreement. Correlation between the stage of the disease and the duration of symptom before MRI was calculated using Kruskal–wallis test.

**Results:**

Various degrees of CET lesions were demonstrated in this population (Stage I-17, IIA-7, IIB-22 and III-29). Intra-observer agreements of MRI staging were substantial to satisfactory. Inter-observer agreements were moderate to substantial. There was no significant correlation between the disease stage and the patient age or the duration of symptom before MRI.

**Conclusion:**

Our MRI classification has emerged as one of the most reliable methods to define stages of chronic lateral epicondylitis. At the end, we have suggeted a clearer direction for understanding the disease pathology as well as an appropriate management protocol for each stage of the disease in line with the recent body of literature.

## Background

Lateral epicondylitis or tennis elbow is a common problem in orthopedic patients, whose presentation chiefly involves lateral side elbow pain. The overall incidence rate was 4.5 per 1,000 in the year 2000 and had dropped to 2.4 per 1,000 in the year 2012 [1]. The disease pathology encompasses chronic inflammation and degeneration of CET origin, especially extensor carpi radialis brevis (ECRB), which has been hypothesized to be caused by micro-repetitive trauma from overuse and excessive load [2]. Tendon degeneration is the main cause of progressive tendon tears from humeral attachment [3], which produces pain and disturbs the elbow function during activities of daily living. The current treatment of lateral epicondylitis starts with options of conservative treatment such as activity modification, physical therapy and anti-inflammatory medicine [4]. However, if the patient fails to respond to such conservative management, the next line of treatment will involve a more invasive approach, namely platelet rich plasma injection (PRP) to the pathologic tendon [5–7], or ECRB debridement of the degenerative tendon in the final stage of this disease [8]. The current choices of treatment vary depending on clinician experience regarding the estimation of the severity of the disease. However, as for the current standard treatment, no consensus classification is available for guiding the treatment by the disease severity. Without a proper course of treatment, the patients will have a higher risk of further tendon degeneration and progressive tendon rupture.

To evaluate the degree of CET injury, tendon interruption was detectable in both ultrasound and MRI settings. The ultrasound demonstrated the anechoic or hypoechoic fluid filling the gap between tendon fragments [9], while the MRI demonstrated the bright T2 signal filling the gap. However, tendon echogenicity in the ultrasound depends on the angle of the transducer as the operator is required to indicate tendon pathology. MRI of elbow can provide the information of the tendon status such as tendinosis, the degree of tendon tear and the location of the tendon origin site at the humeral attachment, which are related to histopathologic findings. Moreover, MRI is an objective tool of investigation with good inter and intra-observer reliability [10–12], being particularly helpful for surgical planning. Hence, MRI can precisely evaluate the extent of the CET lesion compared to the ultrasound approach [13, 14].

From the literature review, the tendinopathy grading through MRI has already been discussed in the rotator cuff tendon [15] and Achilles tendon [16]. There are few MRI grading studies looking into the degree of CET injury, as described by the combination of various variables such as the gap of tendon, the thickness of tear and the degree of tendinosis [17, 18]**.** Nevertheless, the complexity of grading still offers no consensus to clinicians, who resort to MRI information for establishing treatment decisions. Therefore, the objective of this study is to propose a simplified MRI classification, which can facilitate the evaluation of tendon severity, and determine its interrater validity. Finally, the updated literature review of treatment suggestions with reference to the MRI stage is presented in the discussion part.

## Methods

The study was approved by the Institutional Review Board of Ramathibodi Hospital, Mahidol University (COA.MURA2021/894). The study was conducted in the orthopedic department and radiology department, Ramathibodi Chakri Naruebodindra Hospital. The study was conducted according to the guidelines of the Declaration of Helsinki.

### Data collection

We retrospectively reviewed 75 consecutive MRI elbow studies in patients with clinical presentation of chronic lateral epicondylitis.

Patients with a history and clinical diagnosis of high energy elbow trauma, osteoarthritis of the elbow joint and tumor were excluded from the study.

The sample size was estimated by setting the intraclass correlation coefficient (ICC) to 0.4 with the power of 90%, Alpha 0.05, Observation per subject 3. The calculated minimum subject was 23 MRI elbow studies. In this study, we registered 75 MRI studies in total to increase the reliability of the research.

All subjects underwent an MRI scan of the affected elbows using a 3-Tesla MR system (Phillips healthcare) with a dedicated surface coil employed. Examination was performed in the supine position with the elbow extended with the palms in supination. The affected arms were placed as close as possible to the center of the MRI tunnel to obtain high-quality images. Parameters of Coronal T-2 weighted fat-suppressed (T2FS) MRI sequences, which were used for interpreting the classification, are provided in Table 1.

**Table 1:** Parameters of Coronal T-2 weighted fat-suppressed MR sequence

Plane	Sequence	TR(ms)	TE(ms)	ETL	Matrix	BW(Hz)	FOV(mm)	Thickness(mm)	Gap(mm)
Coronal	T2FS TRFSE	3273	80	10	320 × 251	174.8	160	3	3

*FSE* Fast spin echo, *FS* Fat suppressed, *TR* Repetition time, *TE* Echo time, *ETL* Echo train length, *BW* Bandwidth, *FOVE* Field of view

All MR images were analyzed separately by one musculoskeletal radiologist and two upper extremity orthopedists, who were blinded to all clinical data and were unaware of the severity of disease. Each interpreter assessed the images twice, at least 3 weeks apart.

A staging system was devised to measure the severity of tendon injury at the lateral epicondyle. The thickness tear was measured in percentage by the Coronal T-2 weighted fat-suppressed on the image with the maximum degree of tear from the inside to the outside tendons.

Tendinosis and tear were distinguished from each other by the presence or the absence of tendon rupture from bony origin. Tendinosis refers to the intra-tendinous abnormal signal change, while the presence of the abnormal bony signal in the lateral epicondyle only at the CET origin area was described as a bone bruise lesion or bone edema.

**Fig. 1:**
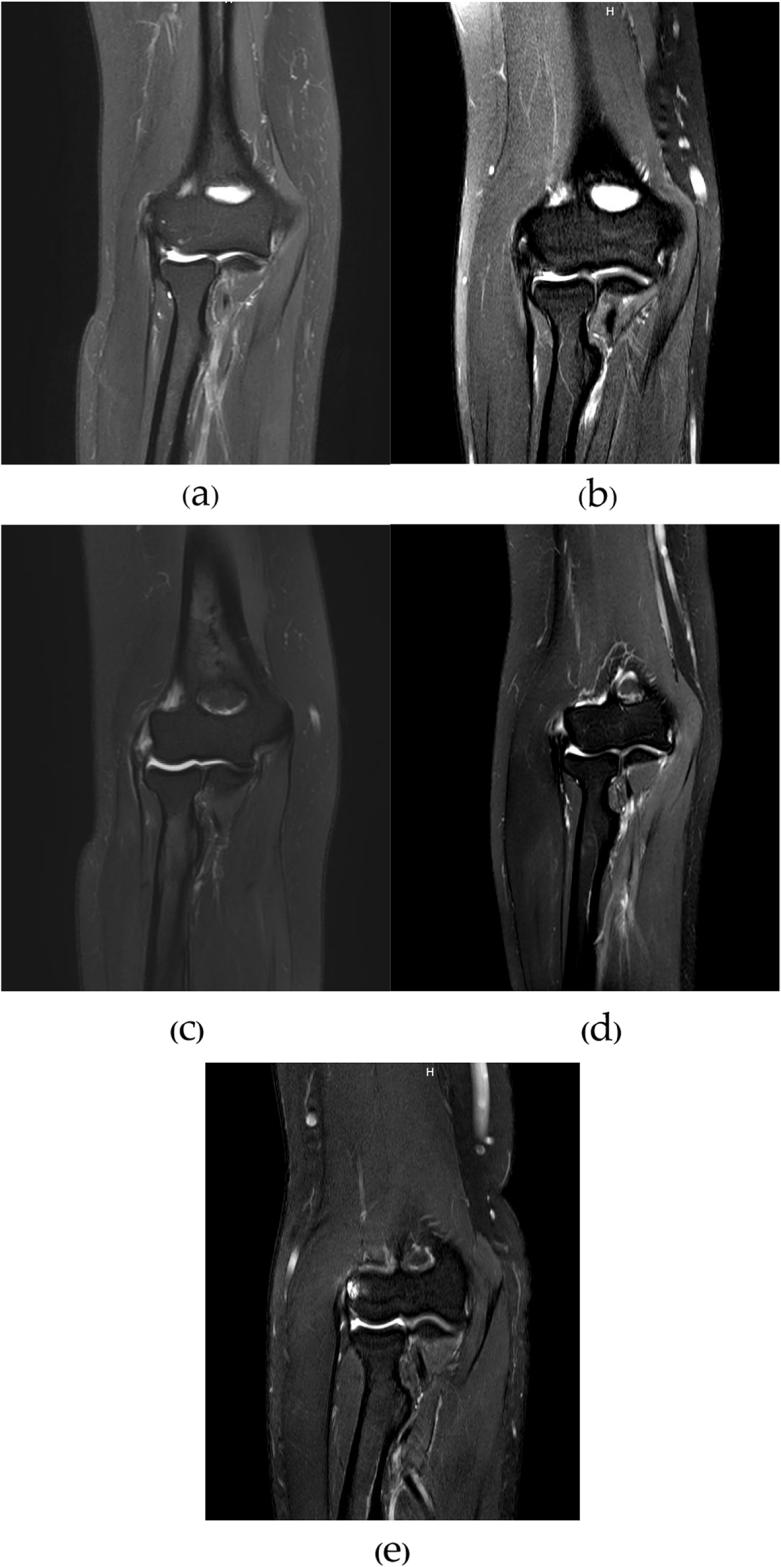
MRI illustration for the degree of common extensor tendon injuries. The measurement in Coronal T-2 weighted fat-suppressed images shows the maximum degree of tear from the inside to the outside CET (**a**) type I-tendinosis (**b**) type IIA-partial thickness tear less than 50% (**c**) type IIB-partial thickness tear more than 50% (**d**) type III-full thickness tear (**e**) type III-bone bruise lesion

The staging was classified to the following stages: Stage I tendinosis, Stage IIA (partial thickness tear less than 50 percent), Stage IIB (partial thickness tear equal to or more than 50 percent), Stage III (complete common extensor or partial tear with bone edema). Table 2 and Fig. 1 describe the MRI findings and classification.

**Table 2:** MRI classification for the common extensor injuries

Stage	Common extensor tendon	Description
I	Tendinosis	Intra-tendinous abnormal signal change
IIA	Partial thickness tear < 50%	Abnormal tendon signal starting from the bony origin less than 50 percent of the thickness
IIB	Partial thickness tear $\ge$ 50%	Abnormal tendon signal starting from the bony origin equal to or more than 50 percent of the thickness
III	Full thickness tear or Bone edema	Whole thickness abnormal tendon signal change of the CET or the presence of abnormal bony signal at the lateral epicondyle

Measurement of the CET thickness tear in Coronal T-2 weighted fat-suppressed on the image depicting the maximum degree of tear from the inside to the outside CET

### Data analysis

MedCalc Statistical Software version 19.2.6 (MedCalc Software bv, Ostend, Belgium; https://www.medcalc.org; 2020) was used for data analysis. The agreement values for MRI classification were calculated. An inter and intra-observer reliability between interpreters was analyzed by the linearly weighted Fleiss Kappa statistic, to evaluate the inter-rater agreement and consistency between two orthopedists and one radiologist. The classified MRI staging was calculated (Stages I, IIA, IIB, III). Kappa values from 0.41 to 0.60 were considered fair/ moderate, 0.61 to 0.80 substantial, and > 0.81 satisfactory [19].

In the next step, the association among the stages and the duration of symptoms before the MRI examination were analyzed by using Kruskal–wallis test. The association was considered significant at *P* < 0.05.

## Result

Out of 75 MRI studies, the number of affected elbows was 28 for the Lt. side and 47 for the Rt. Side. There were 57 women and 18 men with the mean age of 51.4 years (range: 34–73 years) The average total duration of symptoms prior to the MRI examination was 8.6 months. None of the patients underwent corticosteroid injection within 3 months of MRI examination. No patients had received prior surgical treatment. All the patients showed MRI signal change at the lateral epicondyle in various stages. 17 patients had evidence of tendinosis as described in Stage I. 8 patients had partial thickness tear < 50 percent (Stage IIA). 23 patients had partial thickness tear > 50 percent (Stage IIB). 28 patients were classified into Stage III (26 patients for complete thickness tear and 2 for bone bruise lesion).

The average intra-observer agreement was 76% for staging the severity of tendon injury. The weighted kappa values for intra-observer reliability were 0.93 for the musculoskeletal radiologist, 0.72 and 0.64 (*p* < 0.001) for two orthopedic surgeons, respectively. The indicated scale was satisfactory for the radiologist and substantial for the orthopedists (Table 3).

**Table 3:** Intra-observer reliability for grading the degree of common extensor tendon injury

Interpreter	Linearly weighted Kappa	Standard error	95% CI
Musculoskeletal radiologist	0.93	0.04	0.84496–1.00000
Senior upper extremity orthopedic surgeon	0.72	0.06	0.59576–0.84948
Upper extremity orthopedic fellow	0.64	0.06	0.50986–0.77608

The weighted Kappa values for inter-observer agreement were 0.40(*p* < 0.001) between the senior upper extremity orthopedist and the musculoskeletal radiologist and 0.62(*p* < 0.001) between the senior orthopedist and the orthopedic clinical fellow. Indicated faired/moderate agreement between radiologist and orthopedic, substantial agreement between 2 orthopedic surgeons (Table 4).

**Table 4:** Inter-observer reliability for grading the degree of common extensor tendon injury

Interpreter	Linearly weighted Kappa	Standard error	95% CI
Musculoskeletal radiologist and orthopedic surgeon	0.40	0.06	0.28027–0.51973
Two orthopedic surgeons	0.62	0.07	0.47965–0.77654

The median ages of the patients were 52.7(34–73),48.8(37–69),52.8(39–64) and 52.1(42–67) years for Stages I, IIA, IIB, III, respectively. The correlation between the staging and the patient age calculated by ANOVA was not statistically significant (*P*-value > 0.10). The median durations of symptoms before the MRI examination were 44 (24–150), 43 (24–104), 55 (24–156) and 43 (24–104) weeks for Stages I, IIA, IIB, III, respectively. The association among the staging and the duration of symptoms before MRI calculated by Kruskal–Wallis test [20] was not statistically signification (*P*-value was 0.24).

## Discussion

In this study, the stages of tendon injury were classified by the MRI of ECRB tendon depending on the tendon disease pathology. Tendon injury starts with tendinosis (non-inflammatory tendon degeneration), followed by partial thickness tear, which commonly appears as the tear progresses from the weaker junction with the bone to the tendons located outside [3]. When the tear size grows to around more than 50% of tendon thickness, the tendons cannot heal by themselves because the remaining tendons are unable to tolerate the traction force from the extensor, as seen in the high grade partial tear of the rotator cuff muscle [21, 22]. The ending result is full thickness tendon tear. In some patients, the remaining sites of tendon injury create abnormal traction force to the bony attachment, leading to bone marrow edema [23] which can create pain, detected by the presence of bone bruises in MR images.

To evaluate the tendinopathy, both T2w and T1w sequences can be useful. However, T2w imaging can clearly differentiate the torn tendon from the normal tendon by the bright T2 signal [24] (fluid or granulation), which fills the gap between torn tendon fragments, while the T1w sequences cannot clearly differentiate signal intensity between the torn tendon and normal tendon. Moreover, the T2w sequence has less magic angle (artifact signal) compared to the T1w sequence [25]. Therefore, we exclusively used T2w imaging.

In addition, we used only coronal images to measure the percentage of the tear tendon. An axial image of the tendon can help better differentiate the percentage of the tear. However, in the staging system we intended to distinguish the terms of tendinosis from the tendon tear by the presence or the absence of tendon rupture from the bony origin. Tendinosis means the abnormal intra-tendinous signal change. Relying on the axial slice may not identify the spatial relationship between the tendon lesion and the bony origin, as the exact axial location which is related to the same coronal lesion needs a very fine slice. Hence, we did not use the axial image to calculate the tear percentage.

This MRI classification was comparable to the arthroscopic classification and findings obtained from the proximal anteromedial portal, described by Baker et. al for type I-intact capsule, type II-linear capsular tear and finally type III-complete capsular tear [26, 27]. Stage I tendinosis is comparable to the intact capsule, Stages IIA and IIB partial tears to the linear capsular rupture, and Stage III full thickness tear to the complete capsular tear.

The results demonstrated various degrees of CET injury, representing the appropriate demographic distribution in this population. No significant correlation was found between the disease stage and the patient age. In addition, there was no significant association among the disease stages and the duration of symptoms, suggesting that the severity of tendon injury may be affected by multifactorial variables such as basic tendon quality of the patient, habits of extensor usage, previous treatments, and other time-independent factors.

With regard to the agreement of this MRI classification, the musculoskeletal radiologist had the highest kappa agreement level for the intra-observer reliability (satisfactory), followed by the senior upper extremities orthopedist and the upper extremities clinical fellow, respectively. The implication is that the radiologist performed the MRI reading with higher precision than the orthopedists did (substantial), and the more experienced orthopedist may be more precise than the junior orthopedist was.

In terms of inter-observer reliability, the agreement level between the orthopedists (substantial) was higher than between the orthopedists and the radiologist (fair/moderate). This could mean the orthopedists tended to determine the severity of tendon injury from the same perspective and criteria as surgeons. Moreover, the orthopedists and the radiologist may differ in idea or technique employed to classify the cases, especially how to choose which MRI slice was the most appropriate and representative of the severity of the disease. These could result in and explain the difference in the agreement level. Nevertheless, MRI is not the gold standard for establishing the diagnostic degree of tendon injury like arthroscopic diagnosis. Therefore, further studies aiming to compare severity levels between this MRI classification system and arthroscopic findings will help to determine the accuracy and precision of this MRI classification.

Here, we recommend treatment of lateral epicondylitis according to the severity of the disease. In Stage I injury, or tendinosis, the degeneration of tendon cells from micro-repetitive trauma is the cause of pain. In this stage, conservative treatment and biologic therapy such as activity modification, exercise, physical therapy, ultrasound, shockwave and injection of platelet rich plasma(PRP) can be used to reverse tendinosis pathology to the healthy tendon [28]. It is however suggested that corticosteroid injection is to be avoided because it may aggravate tendon degeneration [5].

In Stage IIA partial thickness tear of the tendon less than 50 percent, small tendon tears start to develop, while the remaining tendons still tolerate the force of the forearm extensor. Thus, tendon lesions have a chance to be reversed and undergo self-healing. In this stage, biologic therapy using PRP injection to stimulate tendon healing can play a beneficial role [5–7].

In Stage IIB partial thickness tear of tendon more than 50 percent, a severe degree of tendon ruptured has occurred. Mechanical pain is generated from the remaining tendons, which cannot tolerate to the traction force by the forearm muscle. Progressive tendon tears usually occur under these circumstances. Biologic treatment can be attempted without significant harm to the patient, but surgical intervention is advisable at this stage. The rationale of treatment is translated into two methods. The first method is to stimulate the tendon healing by mechanical intervention such as ultrasound-guided percutaneous tenotomy [29–31]. The other option is to surgically remove the remaining ECRB tendon, which is the pain generator as a result of tendon debridement. Both arthroscopic or open techniques can be performed to achieve treatment goals. However, the arthroscopic technique may have superior ability to access and manage the co-existent intra-articular pathology, compared to the open technique, which has to violate the extensor aponeurosis to gain more joint assessment [27].

In Stage III full thickness tear or partial thickness tear with bone edema, with an irreversible high grade tear, aggressive surgical treatment by open/arthroscopic debridement is recommended [26, 32, 33]. Then after EBRB debridement, a therapeutic option is side-to-side repair alone or repair with the bone anchor to the humeral origin [34, 35].

And in the case of pain from bone edema lesion, decortication (Nirschl procedure) [36] to the bleeding bone can be used to stimulate healing of the bone lesion after tendon debridement.

Our treatment guidance has its foundation on a basic science theory and the degree of tendon tear from MRI findings. However, the clinical information of the patients such as the patient age, the onset, the duration of symptoms and the severity of pain, should be incorporated as part of the treatment decision-making process. Although, this classification constitutes a valid proposal for staging chronic lateral epicondylitis, additional work remains to be carried out and should be performed to determine the accuracy of this imaging test for diagnosing the severity of tendon injury, and to evaluate the result of treatment according to each stage of this disease.

## Conclusion

Our MRI classification of chronic lateral epicondylitis is a valid system for determining the severity of extensor tendon injury. Suggestions of treatment according to the literature review for each stage of the disease should be evaluated for the results.

## Data Availability

The datasets used and/or analyzed during the current study are available from the corresponding author upon reasonable request.

## Abbreviations

CET: Common extensor tendon
CI: Confidential interval
ECRB: Extensor carpi radialis brevis
ICC: Intraclass correlation coefficient
MRI: Magnetic resonance imaging
PRP: Platelet rich plasma
